# Efficacy of an ocular bandage contact lens for the treatment of dry eye after phacoemulsification

**DOI:** 10.1186/s12886-018-1023-8

**Published:** 2019-01-09

**Authors:** Xiaofan Chen, Rongdi Yuan, Min Sun, Xiao Chen, Sen Lin, Jian Ye, Chunlin Chen

**Affiliations:** 1 Department of Ophthalmology, Xinqiao Hospital, Third Military Medical University, Chongqing, 400037 China; 2 Department of Ophthalmology, Research Institute of Surgery & Daping Hospital, Army Medical University, Chongqing, 400042 China

**Keywords:** Bandage contact lens, Phacoemulsification, Cataract surgery, Dry eye

## Abstract

**Background:**

To evaluate the safety and efficacy of a bandage contact lens for alleviating dry eye discomfort after phacoemulsification.

**Methods:**

In this prospective, controlled study, 60 age-related cataract patients with mild Meibomian gland dysfunction (MGD) were randomized to treatment with an ocular bandage contact lens (BCL) (*n* = 30) or to an untreated control group (n = 30) after phacoemulsification and intraocular lens implantation. The Ocular Surface Disease Index (OSDI) questionnaire, evaluation of subjective symptoms and evaluation of the best-corrected visual acuity (BCVA) were conducted preoperatively and postoperatively on days 1, 7, 14, 30 and 90. The tear breakup time (TBUT), Schirmer test with anesthesia, and fluorescein staining scores were measured preoperatively and postoperatively on days 7, 14, 30 and 90.

**Results:**

There were no significant differences with respect to the BCVA between the groups at any time point. For the comparison of the OSDI, subjective evaluation scores, TBUT and fluorescein staining, statistically significant improvements were noted in the BCL group, especially on days 7 and 14 (*P* < 0.001, P < 0.001; *P* = 0.031, *P* = 0.009; *P* = 0.021, *P* = 0.028; and P = 0.03, *P* = 0.032, respectively). The Schirmer test results did not significantly change postoperatively.

**Conclusions:**

A BCL can improve tear film stability and lessen dry eye discomfort immediately after phacoemulsification.

**Trial registration:**

Current Controlled Trials ChiCTR-INR-16008863 (Date of registration: 20 July 2016).

## Background

Phacoemulsification is one of the most effective ophthalmic operations today. Although most patients obtain excellent postoperative distance visual acuity, some complain of continued ocular discomfort, such as a burning sensation, fatigue, foreign body sensation and other dry eye symptoms, which greatly reduce visual quality [1–5]. Although many factors can cause dry eye after cataract surgery, Meibomian gland dysfunction (MGD) is one important cause that cannot be ignored [6]. The incidence of MGD is related to age, race, and sex [7]. According to reports from the United States and Japan, unhealthy Meibomian glands are present in 20 to 55% of the population, and in Asia, the incidence rate of MGD in persons over the age of 60 years is 46.2 to 69.3% [8, 9]. Because most elderly patients with MGD exhibit only mild symptoms of dry eye, the treatment of Meibomian glands is often ignored by doctors and patients. After cataract surgery, some patients experience severe dry eye symptoms due to decreased Meibomian gland function, ocular surface inflammation [10], and other causes, which may aggravate the visual symptoms and decrease patient quality of life.

Bandage contact lenses (BCLs) are primarily used for corneal diseases, and following ocular surgery, the clinical application of BCLs is typically focused on corneal lesions [11, 12], cornea refractive surgery and corneal transplantation [13–15]. These bandages have also been used in cataract surgery, but these studies focus on protecting the corneal wound [16, 17]. However, few studies have assessed the efficacy of BCLs in the relief of dry eye discomfort after phacoemulsification, especially among MGD patients.

The purpose of this study was to evaluate the efficacy of BCLs in alleviating dry eye signs and symptoms after cataract surgery, especially for age-related cataract patients with MGD.

## Methods

Patients with age-related soft-nucleus cataract and suffering from mild-to-moderate Meibomian gland dysfunction (MGD) were enrolled, and informed consent was obtained from all subjects before participation in the study.

Exclusion criteria included patients with severe MGD or other comorbid ocular diseases such as ocular allergies, trachoma, pemphigoid, chemical injury, thermal burns, radiation injury, eyelid anomalies such as entropion and ectropion, blepharospasm, hypophasis, trichiasis and ptosis, Sjögren syndrome, glaucoma or ocular hypertension, lacrimal disease, macular disease, history of any ocular operation or ocular injury, and constant use of topical ocular drugs before surgery.

## Randomization and treatment administration

The study was performed in accordance with the tenets of the World Medical Association of Helsinki and was approved by the Daping Hospital Institutional Review Board, Chongqing, China. The study duration was from August 2016 to February 2017. The clinical trial registration number is ChiCTR-INR-16008863.

All patients underwent standard phacoemulsification through a 2.8-mm clear corneal temporal incision and intraocular lens (Akreos Adapt AO, Bausch & Lomb) implantation by the same surgeon. Prior to surgery, patients were randomly assigned to the intervention group, which would receive an adherent ocular BCL after surgery, or to the control group that would not. The surgeon was blinded to the allocation of all patients until the surgery was completed, at which time the surgeon applied the BCL (PureVision; Bausch & Lomb Inc., Rochester, NY) if indicated. Patients were also masked to their group allocation during the study until the BCL was removed one week later. Postoperatively, all patients received the same treatment, including administration of topical anti-infectives, corticosteroids and nonsteroidal anti-inflammatory drugs.

Patients were evaluated preoperatively and postoperatively on days 1, 7, 14, 30 and 90. On each visit, the following evaluations were conducted in sequence: Ocular Surface Disease Index (OSDI) questionnaire, subjective symptoms, best-corrected visual acuity(BCVA), tear film breakup time (TBUT), corneal fluorescein staining and Schirmer test with anesthesia.

We used the standard OSDI questionnaire and a patient subjective symptom evaluation form to evaluate dry eye symptoms as described in previously published papers [18, 19]. In the OSDI questionnaire, 12 questions were included, and each was rated from 0 to 4; the overall score was derived after evaluation.

The subjective symptom evaluation related to 11 ocular symptoms (foreign body sensation, photophobia, itching, eye pain, eye heaviness, eye fatigue, eye discomfort, eye secretions, blurred vision, dry eyes, and tearing), and each symptom was graded as follows: 0, never; 1, occasional; 2, often; and 3, always.

The BCVA was measured by the same optometrist at each visit.

TBUT was performed to assess tear film stability. Briefly, a single fluorescein strip (Tianjin Jingming New Technological Development Co, Ltd., Tianjin, China) was placed in the conjunctival sac of the eye after instilling a drop of normal saline, the patient was asked to stare straight ahead without blinking, and the time from the last blink to the first appearance of a dry spot was recorded.

Corneal fluorescein staining was performed as previous described. The corneal surface was divided into four regions, and each region was scored as follows: 0, no staining; 1, between one and three dots; 2, less than five dots; and 3, bulk or strip staining. The four regions scores were then added to obtain a final score for the eye.

The Schirmer test was performed by inserting a test strip (HESSEN Biotechnology, Inc., Beijing, China) in the mid-lateral portion of the lower eyelid margin with topical anesthesia. The extent of wetting was measured after 5 min.

All measurements were performed by the same ophthalmologist preoperatively and at days 1, 7, 14, 30 and 90 postoperatively, except for the day 1 postoperative check of the TBUT, corneal fluorescein staining, and the Schirmer test.

## Statistical analysis

Descriptive statistics for continuous data are reported as the mean ± SD. Quantitative data were compared by using the t-test (normal distribution) and the Wilcoxon rank sum test (non-normal distribution) between the two groups before and after surgery. The level of significance was set at *P* < 0.05. Statistical analysis was performed using SAS (version 13.0).

## Results

### Demographics

Sixty-four patients were enrolled at the beginning of the study. Four patients discontinued the study because of intolerance to the BCL. If patients underwent surgery on both eyes, only the first eye was evaluated for this study, i.e., 60 eyes of 60 patients were included in the study. The average age was 61.10 ± 3.96 years (range, 54–68 years; 32 men) (Table 1).

### Visual acuity

There was no difference in the BCVA between the two groups at baseline or at follow-up visits (*P* > 0.05). However, BCVA improved significantly in the two groups after surgery (Table 2).

### OSDI questionnaire and patient subjective symptom evaluation

**Fig. 1:**
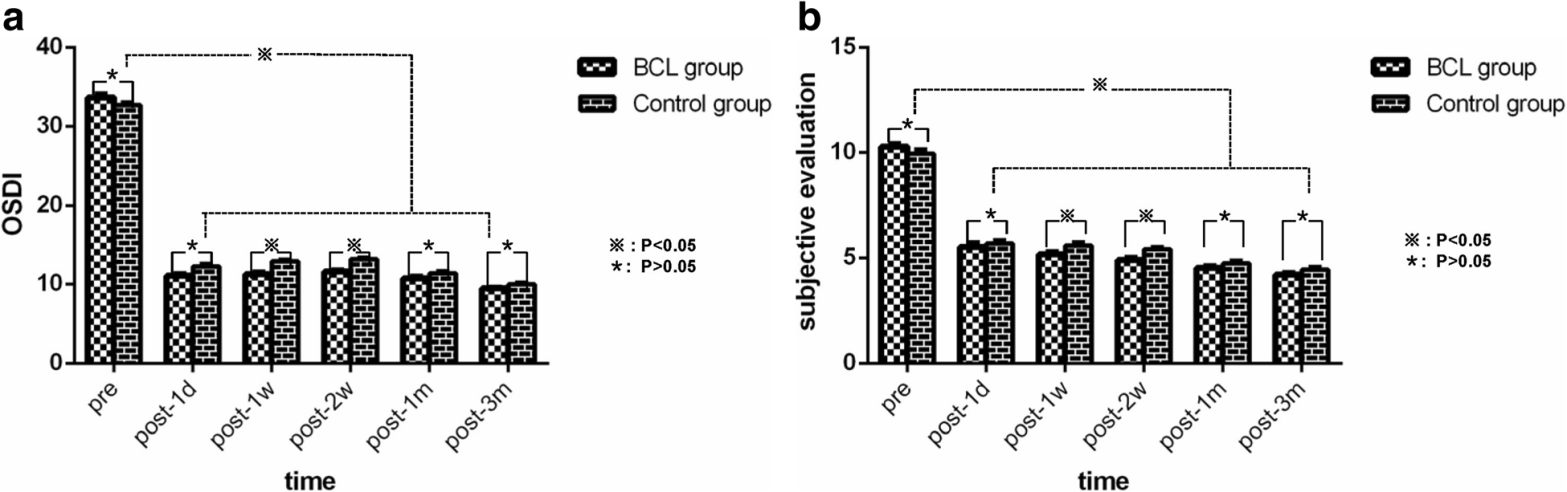
**a** Changes in the OSDI questionnaire objective parameters in the BCL and control groups after phacoemulsification. OSDI scores decreased significantly at each time point from baseline in both groups postoperatively. There were no significant differences between the groups at post-1d, 1 m and 3 m, but significant differences were observed at post-1w and 2w. **b** Changes in the subjective evaluation scores in the BCL and control groups after phacoemulsification. Subjective evaluation scores decreased significantly at each time point from baseline in both groups postoperatively. There were no significant differences between the groups at post-1d, 1 m and 3 m, but significant differences were noted at post-1w and 2w

The differences in the OSDI scores and patient subjective symptom scores (mean ± SD) between the two groups were not significant at baseline (*P* = 0.138, *P* = 0.208). After cataract surgery, OSDI (11.23 ± 1.67 versus 12.87 ± 1.33, P < 0.001; 11.60 ± 1.45 versus 13.17 ± 1.05; *P* < 0.001) and patient subjective symptom scores (5.17 ± 0.79 versus 5.60 ± 0.72, P = 0.031; 4.90 ± 0.80 versus 5.40 ± 0.62, P = 0.009) decreased significantly in both groups at day 7 and day 14. There were no significant differences with respect to OSDI or patient subjective symptom scores between the two groups on days 1, 30 and 90 after cataract surgery. However, both OSDI and subjective symptom scores significantly decreased from baseline at days 1, 7, 14, 30 and 90 in both groups postoperatively (P < 0.001) (Fig. 1a, Fig. 1b)

**Table 1:** Demographic data

	Gender		Age
	Male	Female	
BCL group	15	15	61.8 ± 3.99
Control group	17	13	60.4 ± 3.88
P	0.605		0.173

**Table 2:** Changes in the BCVA in the BCL and control groups after phacoemulsification

		>1.0	≤1.0-0.5	≤0.5-0.3	≤0.3	P
Baseline	BCL group	12	12	6	0	0.759
	Control group	13	11	5	1	
		>0.5	≤0.5-0.3	≤0.3-0.1	≤0.1	P
1 day post-surgery	BCL group	3	5	18	4	0.939
	Control group	4	6	17	3	
7 days post-surgery	BCL group	1	6	18	5	0.908
	Control group	2	7	17	4	
14 days post-surgery	BCL group	1	6	18	5	0.766
	Control group	2	8	17	3	
30 days post-surgery	BCL group	1	5	18	6	0.672

**Fig 2:**
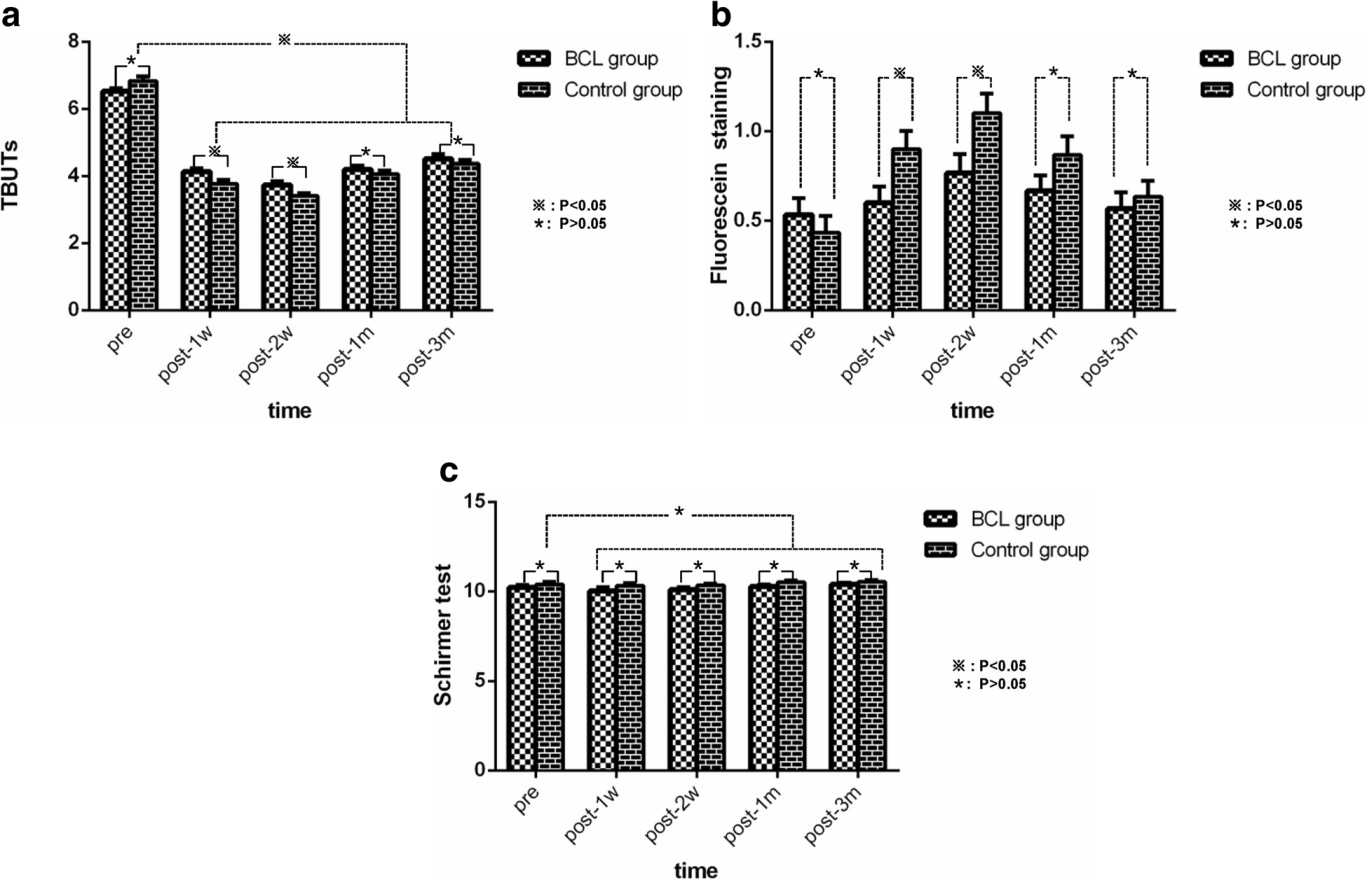
**a** Changes in the TBUT in the BCL and control groups after phacoemulsification. TBUT decreased significantly at each time point from baseline in both groups postoperatively. There were no significant differences between the groups t post-1 m and 3 m, but significant differences were observed at post-1w and 2w. **b** Changes in fluorescein staining in the BCL and control groups after phacoemulsification. There were no significant differences between the groups at post-1 m and 3 m, although significant differences were observed at post-1w and 2w. **c** Changes in Schirmer test in the BCL and control groups after phacoemulsification. There were no significant differences at post-1d, 1w, 1 m and 3 m between or within the groups

.

### Tear breakup time

The difference in TBUT (mean ± SD) between the two groups was not significant at baseline (*P* = 0.062). After cataract surgery, TBUT decreased significantly in both groups, but there was a statistically significant decrease between the two groups on day 7 and day 14 (4.13 ± 0.57 versus 3.77 ± 0.63 s, *P* = 0.021; 3.73 ± 0.64 versus 3.40 ± 0.50s, *P* = 0.028). No differences in TBUT were observed between the two groups on day 30 or day 90 postsurgery. The control group decreased more than the BCL group. However, TBUT significantly decreased from baseline on days 7, 14, 30 and 90 in both groups postoperatively (*P* < 0.001) (Fig. 2a).

### Fluorescein staining

The difference in fluorescein staining scores between the two groups was not significant at baseline (*P* = 0.447). After cataract surgery, fluorescein staining scores increased significantly in both groups, but there was a statistically significant difference between the two groups on day 7 and day 14 (0.60 ± 0.50 versus 0.90 ± 0.55, *P* = 0.03; 0.77 ± 0.574 versus 1.10 ± 0.61, *P* = 0.032). The control group increased more than the BCL group. No differences were observed between the two groups on days 30 and 90.

Compared with baseline, fluorescein staining scores increased significantly on days 7, 14, 30 and 90 in both groups. There was a statistically significant increase from baseline in fluorescein staining scores on day 14 in the BCL group (0.53 ± 0.51 versus 0.77 ± 0.57, *P* = 0.006) and on days 7, 14 and 30 in the control group (0.43 ± 0.50 versus 0.90 ± 0.55, *P* < 0.001; 0.43 ± 0.50 versus 1.10 ± 0.6, P < 0.001; 0. 43 ± 0.50 versus 0.87 ± 0.57, *P* = 0.002) (Fig. 2b).

### Schirmer test

The difference in the Schirmer test (mean ± SD) between the two groups was not significant at baseline or on days 7, 14, 30 or 90 between or within the two groups (*P* > 0.05) (Fig. 2c).

## Discussion

In the clinic, the effects of ocular surface discomfort caused by dry eye after cataract surgery may not be noted by the physician. Conversely, some cases may be diagnosed as viral, bacterial or allergic conjunctivitis and given relevant therapy, thereby inevitably increasing dry eye symptoms and ocular surface anomalies [20].

Eye discomfort after cataract surgery is primarily caused by the development or aggravation of dry eye [1, 21]. Because the majority of patients with cataracts are elderly, ocular abnormalities following surgery are more likely due to decreased tear secretion, poor ocular surface stability, and the ability to resist injury. Thus, paying attention to the possibility of dry eye postsurgery and providing timely and correct diagnoses and therapies are very important.

Dry eye after cataract surgery can be caused by a variety of factors, including surgical incision, preoperative use of anesthesia, mechanical injury and light microscopy stimulation during the operation. Additionally, induced or aggravated MGD is one important cause.

An increasing number of studies have shown that MGD plays an important role in ocular surface abnormalities after various types of eye surgery. Cataract surgery can induce or aggravate MGD [10]. Studies have demonstrated that the function of Meibomian glands can be changed after cataract surgery without obvious morphological changes of the Meibomian glands [6, 10, 22]. Although the exact mechanism of MGD induced or aggravated by cataract surgery is not yet clear, many factors may be involved in this process, including ocular inflammation caused by surgery itself, reduced blink frequency caused by decreased corneal sensation, etc. Cataract surgery may be a trigger to induce or aggravate MGD. However, once MGD is induced or aggravated, MGD will increase the evaporation of tear film, leading to instability of the tear film and excessive evaporation, which may be the cause of worsening dry eye symptoms after cataract surgery.

Currently, bandage contact lenses have been widely applied in ocular surface diseases, especially silicone hydrogel corneal contact lenses. The silicone hydrogel bandage contact lens material itself contains many tiny silicon oxygen channels, which allow molecular oxygen to move freely into the lens. Furthermore, the silicon material also exhibits a good capacity for water absorption. Therefore, the silicon can help lock in water to mimic the lipid layer, thereby reducing tear evaporation and making it suitable for tear hyper-evaporative dry eyes.

To uniformly evaluate and compare the role of corneal bandage contact lenses, the subjects of this study were all cataract patients with MGD. Our results demonstrate that after cataract surgery, nearly all patients had dry eyes. Furthermore, the OSDI increased and the subjective symptom scores decreased significantly after the operation compared with baseline. In addition, the TBUT and FSS both changed significantly. Before the operation, patients did not exhibit typical dry eye symptoms secondary to mild MGD. After the cataract operation, although the patients’ visual acuities improved significantly, the subjective symptom score and TBUT remained unimproved for three months, which indicates that dry eye occurring after an operation cannot be ignored in cataract patients with MGD. After having patients wear bandage contact lenses for one week, we observed significantly increased TBUT and decreased FFS on day 7 and day 14, which suggests that the early application of BCL after cataract surgery can enhance patient comfort and improve the symptoms of dry eye, possibly secondary to promoting corneal wound healing and increasing the stability of the tear film.

No patients complained of redness, eye pain, foreign body sensation or other discomfort. Additionally, no patients exhibited corneal ulcers, corneal neovascularization or other complications, which indicates that wearing corneal contact lenses is safe and reliable after cataract surgery. However, there are some limitations to this study. 1) The sample size is small, and the observation time is short. To better confirm the effect of relieving dry eye symptoms by wearing corneal contact lenses, a larger sample and longer study duration are required. 2) No artificial tears were administered to the control group. 3) No comparison was performed of different time periods of wearing BCL. Despite the abovementioned limitations, we found that cataract surgery can induce or aggravate dry eye symptoms. For patients with cataracts associated with MGD, BCL after the operation could improve tear film stability and reduce dry eye symptoms, though it is perhaps somewhat of a burden for patients.

## Abbreviations

BCL: Bandage contact lens
BCVA: Best-corrected visual acuity
MGD: Meibomian gland dysfunction
OSDI: Ocular surface disease index
SD: Standard deviation
TBUT: Tear breakup time
